# Pickleball for Inactive Mid-Life and Older Adults in Rural Utah: A Feasibility Study

**DOI:** 10.3390/ijerph18168374

**Published:** 2021-08-07

**Authors:** Paige Wray, Callahan K. Ward, Cindy Nelson, Sandra H. Sulzer, Christopher J. Dakin, Brennan J. Thompson, Matthew Vierimaa, Debasree Das Gupta, David A. E. Bolton

**Affiliations:** 1 Department of Home and Community, Utah State University Extension, Monticello, San Juan County, UT 84535, USA; paige.wray@usu.edu; 2 Department of Home and Community, Utah State University Extension, Panguitch, Garfield County, UT 84759, USA; callie.ward@usu.edu; 3 Department of Home and Community, Utah State University Extension, Beaver, Beaver County, UT 84713, USA; cindy.nelson@usu.edu; 4 Department of Kinesiology and Health Science, Utah State University, Logan, UT 84322, USA; sandra.sulzer@usu.edu (S.H.S.); chris.dakin@usu.edu (C.J.D.); brennan.thompson@usu.edu (B.J.T.); debasree.dasgupta@usu.edu (D.D.G.); 5 School of Kinesiology, Acadia University, Wolfville, NS B4P 2R6, Canada; matthew.vierimaa@acadiau.ca

**Keywords:** pickleball, older adults, mid-life adults, exercise adherence

## Abstract

Many diseases, disabilities, and mental health conditions associated with aging can be delayed or prevented through regular exercise. Several barriers to exercise, many of which are exacerbated in rural communities, prevent mid-life and older adults from accessing its benefits. However, recently, a racquet sport named pickleball has become popular among older adults, and it appears to overcome some of these barriers. We conducted a feasibility study to evaluate the impact of a six-week pickleball intervention on measures of muscle function, cognitive function, perceived pain, and cardio-metabolic risk, as well as several psychosocial factors contributing to adherence in sedentary rural participants. Participants improved their vertical jump, cognitive performance, and reported a decrease in self-reported pain, suggesting improved physical and cognitive health across the sample. Participants also reported high levels of satisfaction and demonstrated good adherence over the duration of the study. Perhaps of greatest value was the overwhelmingly positive response from participants to the intervention and follow-up interviews reporting a desire to continue pickleball play beyond the study period. Overall, pickleball appears to be a promising intervention to, (1) elicit functional- and cognitive-related improvements, and (2) motivate mid-life and older adults to adhere to exercise sufficiently long to benefit their health.

## 1. Introduction

A broad range of diseases and disabilities that often accompany aging can be delayed, prevented, or even reversed through lifestyle behaviors, of which exercise is arguably the most scientifically validated [1]. The most concerning of these disorders includes frailty-related issues, which increase the risk of falls (e.g., sarcopenia, dynapenia, and osteoporosis) [2], cognitive decline [3], and cardio-metabolic factors (e.g., heart attack, stroke, and obesity) [4]. Currently, only 23.2% of adults over the age of 18 meet the federal guidelines for physical activity (aerobic and muscle strengthening). This percentage decreases with age [5] and in rural communities [6]. Despite the preponderance of evidence and awareness of the health benefits of exercise, such knowledge has not translated into increased frequency of exercise among the general population. Given that the majority of the population does not currently meet federal exercise guidelines [7], and that exercise compliance rates drop substantially at older ages, maximizing exercise participation and long-term adherence could have enormous benefits to longevity, quality of life, and medical costs for individuals and across the population at large.

While there are many reasons that adults do not engage in regular exercise [8,9,10,11,12,13], the present feasibility study focused on four, all of which are significant barriers to exercise participation and adherence. First, many traditional forms of exercise (such as walking or resistance training) offer limited opportunity for social interaction, which is one of the motivators among mid-life and older adults for engaging in exercise (9). Second, some forms of exercise have prohibitive equipment, travel, or venue expenses (e.g., cycling, racquetball, skiing, Pilates, etc.) [12]. Third, many adults, particularly those who are sedentary, have concerns about injuring themselves while exercising [11]. Finally, exercise often requires considerable commitments of time (e.g., to acquire the skills to play a sport) and effort [10,11]. Collectively, these factors reduce the motivation to exercise and negatively impact exercise adherence [9,10]. Moreover, these barriers can be exacerbated in rural communities for many reasons, including a shortage of athletic facilities, insufficient population to sustain group exercise classes (e.g., yoga or Pilates), a scarcity of qualified coaches or personal trainers, and inadequate infrastructure, such as incomplete sidewalks for running or walking [13]. Indeed, physical activity levels, particularly participation in vigorous-intensity activity, in rural communities tend to be lower than their urban counterparts [14,15]. Therefore, to achieve the long-term participation necessary to prevent or delay age-related disease and disability, an exercise program must address most, if not all, of these four barriers and be accessible to rural communities.

Participation in team sports may provide an ideal way to overcome these barriers while addressing the limitations of traditional solitary exercise programs (e.g., walking, weight training). Sport-based activities are advantageous due to their strong social element, increased cognitive demand, high intrinsic enjoyment, and breadth of physiological systems engaged (e.g., neuromuscular, skeletal, and cardiovascular systems to meet the reactive strength, balance, coordination, and endurance demands) [16]. However, not all sports are equal in their capacity to generate positive health changes. Some sports may be too physically demanding (e.g., rugby), as older adults often cite health or injury concerns as barriers to their involvement in sport and exercise [17]. Other sports may not provide adequate stimulus to elicit broad health benefits (e.g., softball, where much of the time is spent inactive). Racquet sports appear to be an ideal compromise between these two extremes and are reported to have a robust risk reduction for all-cause mortality compared to other sports [18]. While tennis and racquetball are commonly played, they can be too intense and high impact for older adults. In addition, the technical skill required to play these sports may also serve as a barrier to participation.

Recently, a small-court racquet sport named pickleball has become exceptionally popular among older adults because it appears to exhibit advantages that may overcome the aforementioned barriers to exercise [19]. Pickleball holds great promise as a physical exercise intervention for adults of all ages, due to its balance between physical challenge, comparably low injury risk, and ease of play (e.g., short learning curve). In addition, it is highly accessible due to its inexpensive and portable equipment, and it can be played on any moderately sized, hard, flat surface. This combination of accessibility, potential for wide-ranging health benefits, and increased compliance due to its social and enjoyable features suggest that pickleball may offer an effective and low-cost way to improve physical activity levels benefitting the health of mid-life and older adults, particularly those in a rural environment. The rural environment poses unique physical activity barriers [13] which are further aggravated by lack of transportation (to facilities) and limited social support available to rural older adults [12]. Yet, prior studies have examined physical activity promotion mainly in the context of urban settings [20,21], leaving a gap in the evidence base on the effectiveness of interventions in rural communities. 

Therefore, the purpose of this feasibility study was to examine the feasibility of using pickleball as a rural exercise intervention to improve muscle function (strength and power), cognitive ability, perceived pain, and cardiovascular health. In addition, we sought qualitative measures of participant enjoyment to assess the likelihood of adherence. For this study, we identified intervention sites in three non-metro counties [22] designated as non-urban (i.e., rural or frontier) by the Utah Department of Health [23] and as rural health professional shortage areas by the Health Resources & Service Administration (HRSA-HPSA) [24]. While considerable variations exist regarding how rurality is defined [25,26], and consequent under- or over-counting of geographies as rural [27], we adopted the Utah Department of Health and HRSA-HPSA rural designations as most relevant to select sites in the state of Utah with unmet rural healthcare needs. As indicated in Table 1, the selected intervention sites—Beaver, Panguitch, and Monticello—are small population bases (<5000 people) located at least 100 miles away from the nearest large city (>50,000 population). Additionally, in all three sites, the 50 years and older population constituted about one-quarter to one-third of the total population of these places.

We recruited inactive rural mid-life and older adults (50 years and older) at the three sites because they are at the greatest risk of incurring inactivity-related health disorders stemming from low exercise participation rates. A “reintroduction” to exercise could have the greatest positive impact on the health and function of this population. We hypothesized that pickleball participation would improve participants’ muscle function, cognitive function, self-reported pain, and cardiovascular health, and yield high rates of adherence and satisfaction. The overarching aim of this study was to determine the feasibility of implementing this popular modified racquet sport as a way to improve health and function in rural mid-life to older adults, as well as the likelihood of participation adherence.

## 2. Materials and Methods

**Figure 1:**
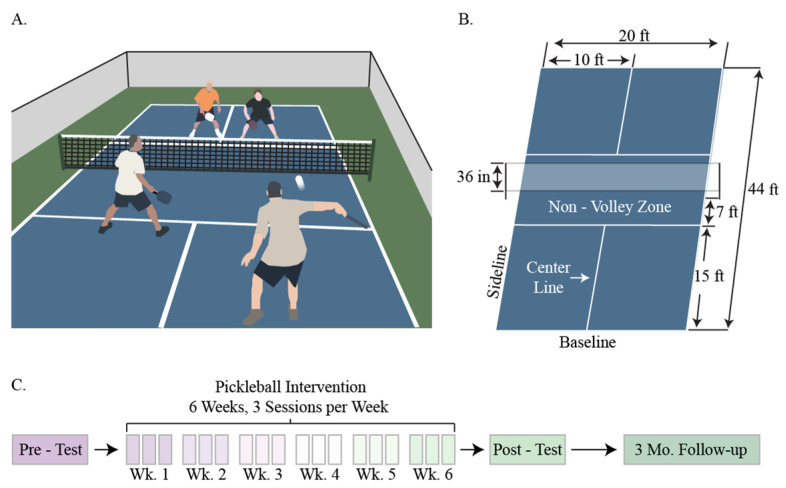
Description of the game pickleball and summary of the experimental design. (**A**). Pickleball is played between two teams of two where, similar to tennis, the goal is to gain points by having the other team be unable to return the ball. (**B**). Pickleball is played on a 44 ft by 20 ft court using a ping-pong-like paddle and a whiffle ball. Pickleball differs from tennis in that the ball must be served underhand and players cannot approach the net to volley (Non-Volley Zone). (**C**). Outline of the experimental design. The study was conducted with physiological measures recorded before and after a 6-week, 3 session per week pickleball intervention. Three months following the post-test, we administered a qualitative follow-up questionnaire examining participants’ perceptions of the intervention. Wk. = week, Mo. = Month.

To determine pickleball’s (Figure 1A,B) feasibility as an exercise intervention in a rural community, participants completed a six-week intervention (Figure 1C). Testing was conducted in 2019 by the Extension faculty at each site, immediately before (pre-test) and within one week following (post-test) six weeks of pickleball play (Figure 1C). Each testing session took approximately two hours to measure muscle function, cognitive function, cardio-metabolic risk, and perceived pain (more details on the specific tests are provided below). In addition, approximately three months after completing the post-test (Figure 1C), follow-up interviews were conducted to identify any lasting psychosocial impact of the intervention to determine whether participants continued to play (i.e., adherence), and what factors influenced the decision to continue play. Extension faculty and staff recruited the rural sample and used Extension’s existing infrastructure to implement the programs in their respective communities.

### 2.1. Participants and Eligibility

We recruited 21 participants between the ages of 50 and 75 (*M_age_* = 58.6, *SD* = 5.8) from across three rural Utah counties (Beaver, Garfield, and San Juan). From this group, 20 completed the study (17 female, 3 male). The average Body Mass Index (BMI) across participants was (*M_BMI_* = 27, *SD* = 4.2), which is classified as ‘overweight’ [30]. Participants were screened for general health status and contraindications to exercise prior to enrollment by local Extension faculty and staff. Individuals were excluded from the study if they had a lower body injury (knee surgery, muscle strain, etc.) within the previous year, had ever had a heart attack, or had any known neuromuscular diseases (e.g., multiple sclerosis). Individuals were also excluded if they participated in more than two hours of structured exercise per week (e.g., weightlifting, running), had played any racquet sports within the previous five years, or had any history of playing competitive racquet sports. Individuals who were advised by their physicians to limit physical exertion were also screened out.

### 2.2. Pickleball Intervention

Participants played pickleball in pairs, three times per week (one hour each session), for a total of six weeks. Extension faculty, staff, and/or student assistants attended each pickleball session to provide instructions, ensure participant safety, teach, and enforce rules. All the pickleball equipment was retained by the Extension offices in each community following the study in order to facilitate the continuation of their pickleball programs.

### 2.3. Outcome Measures

#### 2.3.1. Muscle Function

Muscle strength and power are important predictors of overall health, mortality risk, and functional living capacity [31]. Since grip strength offers a useful marker of overall muscular performance capacity and provides insight into neurological function in general [32], we included it as a simple measure for strength. Grip strength was quantified using a handgrip dynamometer by having participants squeeze a calibrated handgrip dynamometer (Jamar Plus, Patterson Medical, Warrenville, IL, USA) as hard as possible three times, each separated by a 1-min rest, using their dominant hand. Lower body performance was assessed using countermovement jump height, which is an indirect measure of power. Jump performance is an important measure because it is correlated with a number of mobility-related measures [33,34] and is determinative of sarcopenic status [35] in mid- to older-aged adults. Participants performed three countermovement jumps while standing on a jump mat (Just Jump Technologies, Huntsville, AL, USA) using standard procedures (e.g., hands on hips, feet shoulder width apart, no pre-step). The jump mat provides a validated measure of jump height based on flight time [36].

#### 2.3.2. Cognitive Function

Cognitive function is critical to maintaining an independent lifestyle as one ages, and it can be enhanced through physical activity [37]. To quantify the impact of pickleball on cognitive ability, we used a battery of established neuropsychological tests provided by Mindstreams software (NeuroTrax Corp., Newark, NJ, USA). This software provides tests of response inhibition, verbal and nonverbal memory, visual–spatial imagery, and several other aspects of cognitive function. Then, these component measures are combined to calculate a Global Cognitive Score. The Mindstreams software tests correlate with traditional neuropsychological tests such as the Mini-mental State Examination and the Clinical Dementia Rating scale, which have been shown to be valid and reliable measures of cognitive function [38].

#### 2.3.3. Perceived Pain

An important goal of many interventions is to lessen chronic pain, especially since pain is the most common reason for health care visits in the general population [39] and is linked to treatment with higher risk opioid medications [40]. For the present study, the Numerical Rating Scale (NRS) was used to rate pain intensity [39]. Participants were asked to rate their current pain each day on a 0 to 10 scale, where 0 = ‘No pain’ and 10 = ‘Pain as intense as you can imagine’. Although rating pain has potential problems given the subjective nature of one’s perception of pain, the NRS is robust in detecting clinically meaningful changes over time. Scores can be classified as mild (1–4), moderate (5–6), or severe (7–10).

#### 2.3.4. Cardio-Metabolic Risk

To determine the effects of playing pickleball on cardio-metabolic risk factors, we measured blood pressure and resting heart rate.

#### 2.3.5. Psychosocial Factors

Following the intervention period, 15 of the participants agreed to take part in a one-on-one semi-structured interview, which gathered qualitative data regarding participants’ perceptions of the intervention. The interview guide was made up of four sections: Introduction (e.g., Why did you choose to sign up for the pickleball program?), Perceptions of the Intervention (e.g., What was your favorite part of the program? What sort of changes would you make to the program if you were in charge?), Exercise Intentions (e.g., Do you feel as though you have the capability/opportunity/motivation to continue playing pickleball?), and Wrap-Up (e.g., Tell me a little bit about what you found most impactful about the pickleball program.).

### 2.4. Data Analysis

Paired t-tests (2-tailed) were used to compare the pre- and post-intervention measures for each of the following variables: (a) Vertical jump, (b) Grip strength, (c) Blood pressure (systolic, diastolic), (d) Resting heart rate, and (e) Global cognitive score. For the self-reported pain score (i.e., Numerical Rating Scale), a Wilcoxon signed rank test was used (i.e., a nonparametric equivalent of the paired-sample *t*-test). Significance level was set at 0.05. Qualitative interviews were transcribed verbatim and analyzed using summative content analysis [41], which involves the identification of keywords across the interview transcripts followed by latent analysis of underlying meanings and patterns of the participants’ responses.

## 3. Results

**Figure 2:**
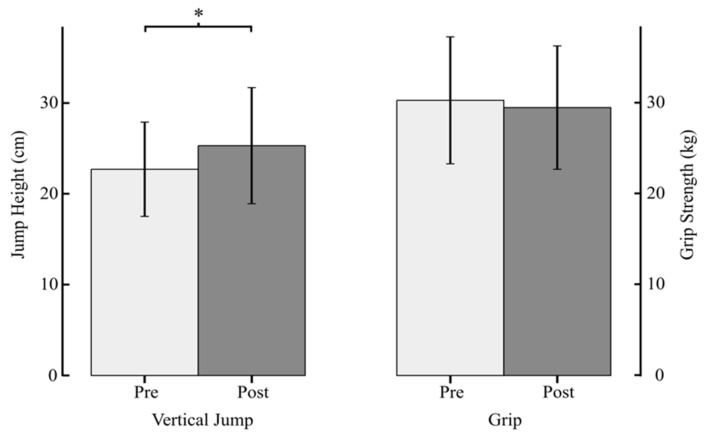
Pre-test and post-test values for jump height and grip strength. Data are means (SD). * indicates significantly improvements from pre-test, *p* < 0.001.

On average, participants attended approximately three-quarters of their training sessions during the six-week intervention period. Specifically, the average attendance across all three rural sites was 13.3 +/− 2.8 sessions out of 18 total training sessions. *Muscle Function*: Vertical jump height significantly increased by 11% (t_19_ = −4.113, *p* < 0.001); however, there was no change in grip strength (t_17_ = 1.106, *p* = 0.284) (Figure 2). *Cognitive function*: There was a small but statistically significant increase in Global Cognitive Score (t_10_ = 2.243, *p* = 0.049) with component scores shown in Table 2. (Note: cognitive testing was conducted on only 11 of the participants due to equipment issues at one of the sites during post-testing). *Perceived Pain*: Self-reported pain revealed a slight but statistically significant reduction from 3 at pre-test to 2 at post-test (z = −2.161, *p* = 0.031). *Cardio-metabolic Risk*: There was no change in systolic (t_19_ = 1.267, *p* = 0.221) or diastolic (t_19_ = 0.018, *p* = 0.986) blood pressure and no change in resting heart rate (t_18_ = 0.519, *p* = 0.610). Outcomes for muscle function, cardio-metabolic risk, and pain are shown in Table 3.

Of the 20 participants that completed the study, 15 completed the follow-up interview with a summary of key themes presented in Table 4. Most participants noted that they initially got involved in the intervention because it seemed fun and because they were encouraged by a peer (i.e., friend, spouse). Fourteen of the 15 participants highlighted that the social aspect (i.e., meeting new friends, deepening existing friendships) was just as (or more) important than the sport itself. In contrast to many other sports, participants liked that pickleball was easy to learn and was not overly strenuous. Several participants noted that they wished that the intervention was more highly structured and intense, citing inconsistent participation and effort among certain individuals. To improve the intervention in the future, participants suggested that sessions should be less frequent but longer in duration, more highly structured (e.g., consistent teams, qualified coaches), and take place indoors where they could participate year-round (note: some sites played on outdoor courts).

Almost all participants described an improved sense of overall well-being (e.g., improved mood, increased energy, higher self-confidence) following the intervention. This change may be partially driven by the fact that post-intervention, several participants found their activities of daily living less daunting and strenuous, and they felt as though their experience in pickleball gave them a foundation for trying out other forms of sport or exercise. Importantly, several participants cited that their improved mood was likely due to both the physical and social aspects of the intervention. Looking ahead, most participants had a positive outlook toward future pickleball participation. Almost all of them found it fun and knew that the equipment was accessible, but “just needed to get around to it”, indicating an intention–behavior gap.

## 4. Discussion

The present feasibility study examined a promising exercise intervention for engaging mid-life and older adults in physical activity. Our main purpose was to explore the feasibility of implementing pickleball in rural settings to address some of the unique barriers faced by rural communities in fostering physical activity, particularly among older adults [12]. In the end, participants improved their cognitive performance and vertical jump, suggesting improved cognitive and physical health across the sample. Participants also reported high levels of self-reported satisfaction with pickleball, and we observed good adherence over the duration of the study. Perhaps of greatest value was the overwhelmingly positive response to the intervention and participant reports of continued play following the cessation of the study provided in the follow-up interviews. Overall, a structured pickleball program appears to be a promising way to motivate this target population to engage in physical activity. This study’s findings are especially relevant given the paucity of evidence on physical activity interventions targeting rural communities [20,21]. One key improvement in our sample following pickleball training was increased vertical jump height. Since vertical jump height is a strong indicator of maximal power output [43], increases in vertical jump height may also be associated with improved fall prevention [44] and enhanced general mobility [32,33,43]. Moreover, leg power capacity has been shown to have a greater influence on a multitude of physical performance measures than leg strength in older adults [45]. Therefore, the present finding, that lower body power-based performance improved by 11% as a result of playing pickleball, is of high significance due to lower body power’s important contribution to functional ability in midlife and older adults. It is interesting to note that increases of approximately 15–16% in vertical jump height have been documented following traditional resistance training and football training (a.k.a. soccer) in older men [46] over the course of a 16-week intervention period. The fact that our group demonstrated an 11% increase despite a much shorter training duration (six vs. 16 weeks, for the present study and the Sundstrup et al. study [46], respectively) suggests that pickleball can significantly improve power output in the lower body to a degree consistent with other reported sport-based interventions.

In addition to a physical challenge, pickleball also poses a cognitive challenge by taxing working memory through the monitoring of participant’s scores and service order during doubles play. Co-training cognitive abilities and physical ability together appears to confer benefits greater than either cognitive training or physical exercise alone [47]. The underlying premise is that physical activity elevates the expression of certain trophic factors in the brain (e.g., fibroblast growth factor messenger RNA) to a level that promotes long-lasting neural changes, leading to greater skill acquisition [47]. Here, we observed a small but significant increase in computerized cognitive test performance, suggesting that pickleball may provide an ideal balance between cognitive and physical challenge at a level that is manageable for older adults.

Physical activity is also known to reduce measures of perceived pain [48,49], and given the current problems with the overuse of pain medications such as opioids [40] but lower access to treatment for opioid use disorder (OUD) in rural communities across the US and Utah [50], exercise interventions could offer an alternative, and perhaps healthier, way to manage or reduce pain. Over the six-week intervention, we observed, on average, a 1-point reduction in median pain scores (pre-test: 3 and post-test: 2). While this reduction in pain is a positive sign, it falls short of a clinically meaningful drop, which is defined as a decrease of 2 points or more on this scale [39]. The tendency toward lower self-reported pain suggests that regular long-term participation in an exercise such as pickleball could potentially reduce self-reported pain and improve quality of life. However, further research is needed to determine if consistent exercise, such as playing pickleball, for durations greater than six weeks or with a gradual increase in exercise intensity can lead to clinically meaningful reductions in self-reported pain.

The benefits conferred by exercise are dependent on long-term continued activity, and therefore, it was important to determine whether participants in this study were likely to continue to play pickleball once the study ended. Of the many factors contributing to exercise adherence, social engagement is one of the more important ones, especially among rural older adults [12]. At the end of this study, participants reported that the social aspect of the intervention figured prominently in their initial decision to participate and that the intervention’s social component contributed to their enjoyment of the intervention. Indeed, several participants noted that the social aspect (e.g., meeting up with friends) was a primary reason that they continued to participate in the intervention. These findings are in line with research suggesting that physical activity programs, with opportunities for social interaction, can reduce loneliness and improve quality of life among mid-life and older adults [51]. Furthermore, recent research highlights a link between pickleball participation, general happiness, and a sense of community among older adults [52], reinforcing the potential of this intervention to address the physical and psychosocial needs of this demographic.

In addition to this preliminary evidence, there are other reasons to extend current research. Pickleball requires quick steps in multiple directions to return an opponent’s ball, so the sport may provide important agility and lateral stability training, which could have a positive impact on balance recovery. Notably, controlling lateral stability is a particular challenge as we age [53]. The rapid changes in posture that occur during play, combined with the cognitive challenges of the game (e.g., selecting the location of a return shot based on an opponent’s location), are ecologically relevant to fall prevention in daily life. Although we did not test balance in this study, pickleball could also positively benefit balance by offsetting age-related balance impairment and therefore potentially reducing fall risk with age. 

### 4.1. Limitations

As with any test of feasibility, the present study has a number of limitations. First, future studies should include a larger sample size and a control group, to account for practice effects from repeated testing or other potential confounds such as the effect of social interaction alone. In particular, the low sample size for our cognitive test poses a risk for type I error; thus, future work using larger samples will be required to verify this result. Sample size may need further adjustment to account for challenges when recruiting participants and retaining them over the study period in low-density rural communities with transportation barriers for older adults. Second, while unintentional, our sample was primarily female (*n* = 17, or 85%), and therefore, our results may not generalize across sexes. In fact, the non-randomized nature of participant recruitment was another study limitation to consider when interpreting present results. Third, the six-week duration of the experiment may have been too short to tax participant adherence. Increasing the duration of the experiment could provide valuable information regarding participant adherence as well as provide more conclusive outcomes for the other measures we chose to record for this study. For example, cardio-metabolic measures often require more than six weeks to observe significant improvements [54]; thus, increasing intervention duration could better resolve the presence and magnitude of improvements in such measures. This would be particularly relevant, since the early stages of our intervention required a familiarization period to learn basic pickleball skills and rules of the game, thus limiting how intense the activity level was in the beginning. As skills grow and the amount of time spent playing at higher intensity increases, there is a reason to believe that the cardiovascular benefits could accumulate over time. It was also noteworthy that grip strength showed no statistically significant changes following our intervention. The inclusion of grip strength into our assessment battery may seem odd given that pickleball would presumably provide little or no training stimulus to upper limb muscles. However, our rationale for including this measure is based on recent evidence suggesting that grip strength offers a window into central nervous system integrity rather than a simple measure of muscle fitness [32]. It is possible that pickleball may fail to cause the systemic changes in neurological function purported to be measured via grip strength dynamometry. Alternatively, this measure may not be sensitive enough to detect small changes or that the brief training period may have been insufficient to stimulate change. Future studies with a longer training duration could help resolve this matter. Lastly, as with any physical activity, pickleball carries an inherent risk of injury [55]. However, this risk must always be measured alongside the many benefits of staying physically active.

### 4.2. Future Directions

One of the most significant findings of this study was a self-report of lowered pain, despite a relatively short intervention. Utah is a state that has struggled extensively with opioid use and mortality, ranking as high as seventh in the nation for overdose deaths [39,54,55]. Finding novel ways to increase exercise engagement in high-risk populations that may be more likely to suffer from chronic pain associated with aging is of dire need, since this can reduce the likelihood of patients seeking out higher-risk pharmacological solutions. This is especially crucial in rural settings where direct education around opioid use is often thwarted due to substantial stigma [56]. Pickleball games amongst aging and older adults is a promising new area of study, especially if future research continues to show statistically significant changes in pain scales, which may lead to an overall reduction in the likelihood of seeking out opioid medications for pain.

Additionally, this preliminary study establishes a foundation to implement pickleball as an exercise intervention on a larger scale to include more communities and a larger number of participants. Notably, such expansion will likely require stratification into different leagues based on age and/or skill level. While we focused on rural communities, given their particular challenge in keeping the local populations physically active, the introduction of pickleball into non-rural settings could confer equal health benefits. In addition, future studies employing a wider range of tests may reveal additional benefits. For example, an important consideration that was unexplored in the present study was pickleball’s impact on balance control. This is important given the increased prevalence of injurious falls with age [57], and notably, the recent attention to consideration of factors leading to falls in mid-life adults [58]. The demand on continual weight shifts and rapid steps during pickleball have a strong theoretical basis for leading to improved reactive balance control and resistance to falls. Therefore, future research could include assessment of balance, particularly reactive balance, given the value of quickly generating a compensatory step response to prevent a fall [59]. Broad metrics of mobility and stamina, such as the six-minute walk test, or the timed-up and go (TUG) test may also offer insight into how this intervention could promote greater function in daily life. Future studies could include more comprehensive questionnaires immediately before and after training to gain greater understanding of behavior change and psychosocial well-being. For all measures, a longer-term follow-up is warranted to gauge retention and determine whether people continue to be physically active following the intervention. The development of a consistent positive lifestyle change would be the ultimate marker of success.

## 5. Conclusions

In this study, we present a promising exercise intervention to overcome barriers to exercise engagement in rural, inactive mid-life and older adults to promote healthier lifestyles. Following a brief training period of six weeks, improvements were noted in vertical jump height, cognitive performance, and self-reported pain. Good program adherence and positive qualitative reports post-intervention indicated pickleball is well-tolerated by this group and could offer benefits in addition to physical health. We purposely focused on rural settings because of the obstacles these communities face engaging in physical activity. However, this intervention could also be implemented in non-rural settings, perhaps with greater ease, given the denser population and additional resources/facilities available in such settings.

## Figures and Tables

**Table 1:** Characteristics of the counties and cities of the three rural study locations, 2019. Population numbers are from the 2019 American Community Survey (ACS) data 5-year estimates [28] (https://www.census.gov/programs-surveys/acs/, accessed on 2 July 2021); area (square miles) for density calculation is from the US Census [29]; study site = the locale where the study intervention was conducted within the respective county, which is the county seat in all cases; distance from urban = distance from the study site to the nearest city with a population >50,000.

County	County Population		Study Site	Site Population and Demographics					Distance of Site from Urban Area (Miles)
	Size	Density (per Square Mile)		Size	Density (per Square Mile)	50–64 Years (%)	65 Years and above (%)	50 Years and above (%)	
Beaver	6517	2.50	Beaver City	3074	459	17.3	18.9	36.2	103.0
Garfield	4998	0.97	Panguitch City	1785	616	16.8	17.7	34.5	108.9
San Juan	15,302	1.96	Monticello City	2604	723	9.8	13.6	23.4	206.0

**Table 2:** Cognitive test results. Raw outcome data are normalized according to age- and education-specific normative data. Then, normalized scores are adjusted to a standard scale with a mean of 100 (standard deviation (SD) = 15). On this scale, 100 represents average performance, a score of 85 is 1SD below average, and a score of 115 is 1SD above average. A Global Cognitive Score is the average of all index scores and serves as a measure of overall battery performance (NeuroTrax data report guide) [42].

Test	Global Cognitive Score	Memory	Executive Function	Attention	Information Processing Speed	Visual Spatial	Verbal Function	Motor Skills
Pre	109.9	106.1	111.3	108.4	112.9	116.4	102.6	111.4
Post	111.8	108.8	110.9	108.7	118.5	118.4	105.1	112.4

**Table 3:** Measures of muscle function, cardiovascular output, and self-reported pain. All values are means ± standard deviations (except ordinal pain scale data reported as median). * = significantly different from the pre-test at (*p* < 0.05).

Outcome Measure	Pre-Test	Post-Test
Vertical Jump Height (cm)	22.7 ± 5.2	25.3 ± 6.4 *
Grip Strength (kg)	30.3 ± 7.0	29.5 ± 6.8
Diastolic Blood Pressure (mm/Hg)	87.5 ± 12.2	87.4 ± 7.3
Systolic Blood Pressure (mm/Hg)	134.9 ± 12.7	129.8 ± 12.8
Resting Heart Rate (bpm)	77.0 ± 15.6	75.7 ± 9.3
Pain (median score 0–10)	3	2 *

**Table 4:** Summary of key themes derived from qualitative interview data.

Interview Section	Themes
Positive aspects of intervention	Socialization (e.g., meeting new friends) Fun and enjoyable Easy to learn and participate, even if you weren’t in the best shape
Negative aspects of intervention	Outdoor facilitates in inclement weather Absent participants made it challenging to organize games Varying levels of intensity and competition among participants
Suggestions for improvement of the intervention	More structure and organization (consistent teams, schedules, etc.) Meet less often, but for longer durations Highly skilled coaches to tech skills and increase intensity levels Indoor facilities for year-round participation
Perceived benefits	Improved overall sense of well-being (e.g., mood, self-confidence) Activities of daily living seemed less strenuous and daunting post-intervention Good springboard to other sports and forms of exercise
Long-term adherence	Most agreed that they had the knowledge and skills needed to keep playing post-intervention Enjoyed that equipment was freely accessible via Extension offices Most agreed that it was fun and want to keep playing, but just need to get around it

## Data Availability

The data presented in this study are available on request from the corresponding author.
